# A characterization of cortisol level and adrenal reservation in human cardiopulmonary arrest: systematic review and meta-analysis

**DOI:** 10.1186/s13643-021-01820-4

**Published:** 2021-10-08

**Authors:** Adeleh Sahebnasagh, Paria Soltani Nejad, Amin Salehi-Abargouei, Mohammad Hossein Dehghani, Fatemeh Saghafi

**Affiliations:** 1 Clinical Research Center, Department of Internal Medicine, School of Medicine, North Khorasan University of Medical Sciences, Bojnurd, Iran; 2 Pharmaceutical Sciences Research Center, School of Pharmacy, Student Research Committee, Shahid Sadoughi University of Medical Sciences, Yazd, Iran; 3 Nutrition and Food Security Research Center, Shahid Sadoughi University of Medical Sciences, Yazd, Iran; 4 Department of Nutrition, School of Public Health, Shahid Sadoughi University of Medical Sciences, Yazd, Iran; 5 Department of Anesthesiology and Critical Care, Shahid Rahnemoun Hospital, Shahid Sadoughi University of Medical Sciences, Yazd, Iran; 6 Department of Clinical Pharmacy, Faculty of Pharmacy and Pharmaceutical Sciences Research Center, Shahid Sadoughi University of Medical Sciences, Professor Hesabi Blvd, Yazd, Iran

**Keywords:** Cardiac arrest, Glucocorticoids, Cortisol level, Adrenal reservation, Systematic review

## Abstract

**Background:**

Cardiopulmonary arrest (CPA) is an urgency, which is associated with high mortality. This systematic review evaluated the relationship between baseline cortisol level and the outcome of resuscitated CPA patients.

**Methods:**

We searched the following databases: PubMed, Scopus, ISI Web of Science, and Google Scholar. Relevant observational and controlled trials were explored from inception by April 2020. The quality of the articles was assessed using the Newcastle-Ottawa Scale (NOS).

**Results:**

Finally, five cohort studies (*n* = 201 participants in total) were eligible for including in the meta-analysis. The results of this meta-analysis showed that although the baseline serum cortisol levels were higher in survivors of cardiac arrest compared with non-survivors, the differences between groups do not reach a significance level (Hedges’ *g* = 0.371, 95% CI, −0.727, 1.469, *P* value = 0.508). Between-study heterogeneity was statistically significant (Cochrane *Q* test: *P* value < 0.001, *I*^2^ = 89.323).

**Conclusions:**

The result of the present meta-analysis was suggestive of a higher baseline serum cortisol levels in survivors of CPA. Future randomized controlled studies with a large sample size will determine the exact relationship between adrenal reservation and the eventual outcome of patients with CPA.

**Systematic review registration:**

PROSPERO CRD42018085468

## Background

Cardiopulmonary arrest (CPA), also known as cardiac arrest, is a severe and potentially fatal condition. Even with successful resuscitation, CPA is associated with high mortality [1]. According to the American Heart Association National Registry of Cardiopulmonary Resuscitation report, the discharge rate of CPA patients is 17.6% [2]. CPA may lead to the following dysrhythmia: ventricular fibrillation, pulseless ventricular tachycardia, and asystole. It should be noted that pulseless electrical activity affects around two million people around the world yearly [3]. CPA is an abrupt loss of cardiac function which can also occur in a person without underlying heart diseases [4]. However, the majority of CPAs are not sudden in the hospitalized patients, as 50‑84% of these patients show the symptoms of hemodynamic instability 1‑6 h before CPA occurrence [2, 5].

Due to the different etiologies of CPA, the symptoms broadly vary, from hypotension and dysrhythmia to changes in consciousness and abnormal breathing [5]. Some patients who experience CPA developed mild to severe neurological and cerebral complications even after discharge from the hospital [6, 7]. Some complications such as myocardial dysfunction, ischemia-reperfusion, and cerebral injury may occur after experiencing CPA. Furthermore, CPA-associated inflammatory responses are primarily responsible for the hemodynamic instability and death in these patients [8]. Similar to severe sepsis and its associated multi-organ failure, the clinical manifestations of a person experiencing CPA include increased production of inflammatory cytokines and release of endotoxins, coagulation abnormalities, and adrenal dysfunction [8–11].

Hypothalamic-pituitary-adrenal (HPA) axis physiology undergoes significant changes during acute and critical illnesses [12]. In stressful situations, the secretion of cortisol increases significantly. Inadequate cortisol production during critical illness can result in hypotension, decreased systemic vascular resistance, shock, and even death [13].

Since CPA is a stressful event, it is expected to detect high levels of stress hormones, such as cortisol, adrenocorticotropic hormone (ACTH), and antidiuretic hormone (ADH) in the blood samples of patients experiencing cardiac arrest. Nevertheless, the existing literature in this field is conflicting. Some previous studies suggested that the activity of HPA axis is relatively suppressed in the cardiac arrest [1, 11, 14–16]. As a result, a relative adrenal insufficiency is observed in CPA patients [1, 11, 14]. Notably, the clinical outcome of patients with higher cortisol levels has been controversial in various studies and has not always been associated with better outcomes. Therefore, this systematic review and meta-analysis aimed to determine the association between the serum cortisol levels and adrenal reserve with the clinical outcome of patients suffered from cardiac arrest.

## Methods

This systematic review was conducted following the Preferred Reporting Items for Systematic Reviews and Meta-analysis (PRISMA) statement, and it was registered at PROSPERO in January 2021 (registration number: CRD42021225420).

### PI(E)CO question

We considered serum cortisol level for the adrenal reserve assessment (exposure) in the patients resuscitated after both in-hospital and out-of-hospital CPA of all etiologies (population), and the rate of survival as the main outcome (outcome) compared to non-survivor patients (comparison).

### Data sources and search strategy

The Medical Subject Headings (MeSH) and other related non-MeSH terms consisted of the synonyms of cortisol and heart arrest were used as keywords. We conducted a systematic search of literature in the following databases: MEDLINE via PubMed (www.pubmed.com; National Library of Medicine), Scopus (www.scopus.com), ISI Web of Science (www. thomsonreuters.com), central register for controlled trials (https://www.cochranelibrary.com/central/about-central), and Google Scholar (www.scholar.google.com). The search was performed until 17 April 2020.

### Inclusion criteria

Inclusion criteria for the present systematic review and meta-analysis were cohort studies, which assessed the following:

- Cohort studies that were conducted among the CPA patients older than 16 years of age
- Studies that assessed the relationship between cortisol level and adrenal reservation in the CPA patients

Duplicate records were automatically removed through the EndNote software. Further duplication removal was done by manual search. Data collection was performed due to the PRISMA flow diagram for reporting systematic reviews and meta-analysis.

### Exclusion criteria

- Studies that were conducted among the patients with a history of corticosteroid therapy within 1 month prior to their referral and those who had received the steroidogenesis-inhibiting agent etomidate, traumatic injury, or terminal illness was responsible for CPA, end stages of chronic diseases like cancer, and steroid use before the CPA.

### Data extraction

We collected the following information from each of the included studies: the first author, year of publication, sample size, the country in which the study was implemented, study design, patient’s demographic features at baseline, and primary/secondary outcomes were extracted from every included study. Then, the data were summarized into a data extraction table (Table 1). Data were extracted and confirmed by two individuals (F.S. and P.S.) independently to ensure their accuracy.

**Table 1:** Characteristics of included cohort studies

Number of study	Author, year	Country	Number, sex F/M^a^	Age (year)	Primary outcome	Secondary outcome	Result
1	Ito, 2004 [17]	Japan	36 (12F/24M)	> 18	Plasma ADH, plasma ACTH, serum cortisol level	-	The serum cortisol levels were significantly higher in the survivors than in the non-survivors (*P* value = 0.029). The plasma ADH and ACTH levels showed no significant difference between the two groups.
2	Hékimian, 2004 [11]	France	33 (4F/27M)	> 16	Serum cortisol level, corticotropin test (250 μg i.v.) for adrenal reserve	Lactate levels	The serum cortisol levels were significantly higher in non-survivors than in the survivors (*P* value = 0.01). Patients who died of early refractory shock had lower baseline cortisol levels than patients who died of neurologic dysfunction (*P* < 0.01).
3	Kim, 2006 [1]	South Korea	30 (15F/15M)	> 16	Cortisol response and determine the RAI after ROSC	Serum cortisol level	The mean cortisol level at baseline were similar among survivors and non-survivors (*P* value = 0.75). RAI (response to corticotrophin test < 9 μg/dL) at initial 12‑24 h and > 24‑48 h occurred in 13 (43%) and 10 (33%) patients, respectively.
4	Tavakoli, 2012 [18]	Iran	50 (23F/27M)	> 18	Serum cortisol Level	-	The serum cortisol levels were higher in the survivors than in the non-survivors, but it was not statistically significant (*P* value = 0. 212). The difference was significantly higher in neurologically survived group in both 5 min and 1 h after ROSC (*P* value = 0.015 and 0.013 respectively).
5	Mosadegh, 2016 [19]	Iran	52 (22F/30M)	> 18	Serum cortisol level	In-hospital death or hospital discharge according to the status of HPA axis function	The serum cortisol levels were higher in the non-survivors than in the survivors, but it was not statistically significant (*P* value = 0.49).

^a^*F/M* female/male, *ADH* antidiuretic hormone, *ACTH* adrenocorticotropic hormone, *μg* microgram, *i.v.* intravenous, *HPA* hypothalamic–pituitary–adrenal, *ROSC* return of spontaneous circulation, *RAI* relative adrenal insufficiency

In case of any disagreements, they discussed with the third author (ASA).

### Quality assessment for individual studies

The quality assessment for cohort studies was conducted using the Newcastle Ottawa Scale.

### Statistical analysis

Mean values for baseline serum cortisol levels and their corresponding standard deviation (SD) for comparing baseline cortisol levels between survival and non-survival groups were used to calculate the bias-corrected standardized mean difference (Hedges’ *g*) and its standard error (SE) to be used as the effect size for meta-analysis. The meta-analysis was conducted using a random-effects model. Sensitivity analysis was conducted to explore the extent to which the summary effects may depend on a particular study or a group of publications. In the case of significant asymmetry in funnel plots, we conducted trim and fill analysis to see if the overall effect changed after establishing the symmetry in the funnel plot [20]. All statistical analyses were done using the Comprehensive meta-analysis software, version 2 (USA). *P* values less than 0.05 were considered statistically significant.

## Results

### Flow of study selection process

**Fig. 1:**
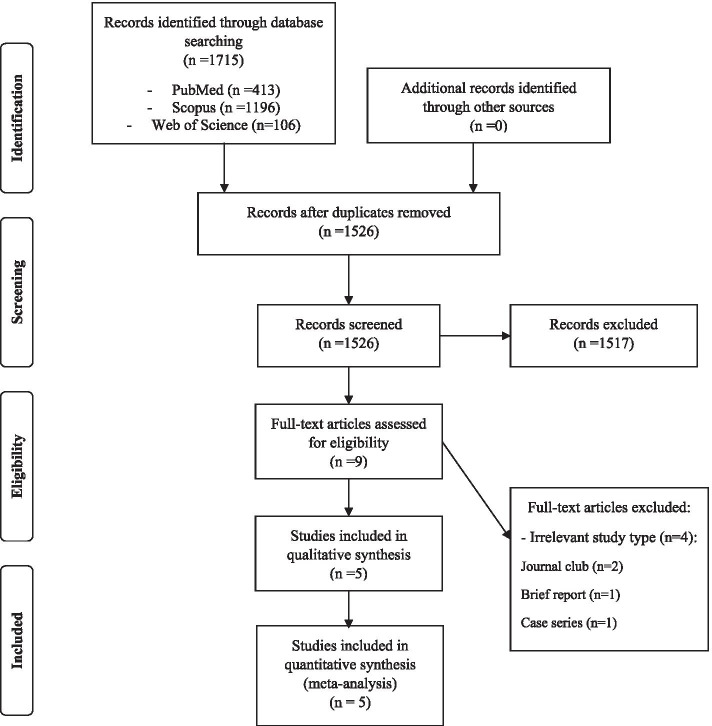
PRISMA flow diagram

The primary search was performed up to April 2020. Our searches identified 1715 records from various sources; five studies with a total of 201 participants met inclusion criteria (Fig. 1). Our search resulted in 413, 106, and 1196 articles from PubMed, ISI web of knowledge, and Scopus databases, respectively. We did not find any new articles by manual search or searching on databases such as Google Scholar. In the next step, 189 duplicates were removed automatically and manually. Finally, searches identified 1526 unique records, of which 1517 articles were potentially irrelevant based on the initial screening. After reviewing the full texts, another four studies were excluded. From these four studies, two studies were journal clubs, and two remaining articles were case series and brief report. Eventually, five cohort studies were included in the systematic review and meta-analysis (Fig. 1).

### Study characteristics

The designs of the included studies were highly heterogeneous. All of the included studies were cohort [1, 11, 17–19]. All studies were published from 2004 and 2016. The duration of studies varied from 3 to 17 months. The only common parameter in all the studies was baseline serum cortisol level. This parameter was higher in survivors than in the non-survivors in two of the studies [17, 18], while the levels were higher in non-survivors in the other three studies [1, 11, 19]. Table 1 represents the characteristics of the included articles.

### Risk of bias and quality of evidence

We used the Newcastle-Ottawa Scale (NOS) to assess the risk of bias in each included cohort studies [21]. Selection, comparability, and outcome were used to summarize the quality of cohort studies. A study can be awarded a maximum of one star for each numbered item within the selection and exposure categories. A maximum of two stars can be given for comparability. The assessment for risk of bias is presented in Table 2. According to the NOS quality assessment scale, all included cohort studies were assessed as having low risk of bias. Data were extracted and confirmed by two investigators (F.S. and P.S.) independently to ensure accuracy.

**Table 2:** Quality of cohort studies

	Selection	Comparability	Outcome
Tavakoli/2012 [18]	★★★	★	★★★
Ito/2004 [17]	★★★	★★	★★★
Hékimian/2004 [11]	★★★★	★★	★★★
Kim, 2006 [1]	★★★	★★	★★★
Mosadegh/2016 [19]	★★★★	★★	★★★

### Meta-analysis

**Fig. 2:**
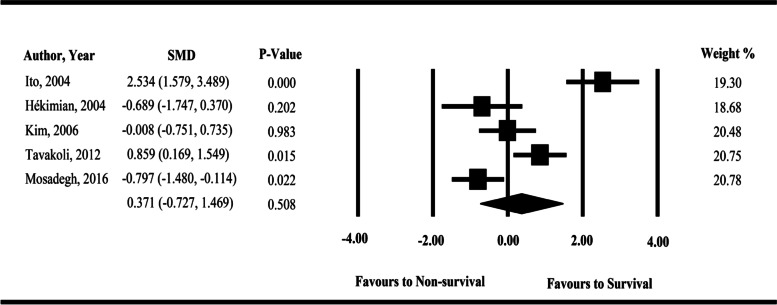
Forest plot of eligible cohort studies which were included in the meta-analysis

The forest plot of included cohort studies for meta-analysis is illustrated in Fig. 2. According to the results of our overall meta-analysis, although the baseline serum cortisol levels were higher in survivors compared to the non-survivors, no significant difference was observed between survivors and non-survivors CPA patients (Hedges’ *g* = 0.371, 95% CI, −0.727‑1.469, *P* value = 0.508). Furthermore, the between-study heterogeneity was statistically significant (Cochrane *Q* test: *P* value < 0.001, *I*^2^ = 89.323).

### Sensitivity analysis

The results of sensitivity analysis showed that none of the included studies has an impact on the rate of survival. In fact, the effect size (ES) for the influence of cortisol level on the survival of CPA patients was robust after removing studies one by one (ES = 0.371; 95% CI, −0.727, 1.469; *P* value = 0.508).

#### Discussion

This systematic review and meta-analysis aimed to explore the relationship between circulating cortisol level and adrenal reservation with eventual outcome of cardiopulmonary arrest. As far as our knowledge, no systematic reviews and meta-analysis has been conducted on the link between cortisol levels and adrenal reserve in cardiac arrest. The results of this meta-analysis indicated that although the baseline serum cortisol levels were higher in the survivors of CPA compared to the non-survivors, the differences between groups did not reach the significance level in CPA patients.

The self-defense responses of the body to stressful events include releasing stress hormones, such as cortisol, ACTH, and vasopressin [22]. Since CPA is the most stressful crisis, it is presumed that the proper and timely secretion of these hormones contributes to the outcome and survival of these patients [23]. Various studies have examined the effects of exogenous administration of these hormones, as therapeutic interventions on the clinical outcome of patients resuscitated after CA [24–27]. As described in prior studies, the hypothalamic-pituitary-adrenal axis is moderately suppressed in this condition. This leads to a diminished release of cortisol from the adrenal cortex. This relative adrenal insufficiency is presented as an inadequate response of the adrenal cortex to the ACTH in victims of CPA [1, 11, 14, 15]. Presumably, the underlying mechanism of this partial insufficiency is ischemia and anoxia of the adrenal glands. Vasopressors compensate for this defect, and cortisol, in turn, improves the vasoconstrictive responses to the effects of vasopressors [28, 29]. Previous studies showed a direct relationship between high ACTH serum levels and relative adrenal insufficiency with poor clinical outcome and mortality rate [1, 15].

Steroids have key roles in suppressing the over-activated systemic inflammation, scavenging the free radicals, and apoptosis. Furthermore, they improve the immune responses, boost cardiac performance, and reinforce the adrenergic responses [30–32]. Moreover, the corticosteroids strengthen and maintain the vascular glycocalyx barrier integrity, which breaks down during cardiac arrest [33]. Therefore, due to mentioned relative adrenal insufficiency and consequent endogenous deficiency of these compounds, it appears that supplemental doses of glucocorticoids may improve the clinical condition of patients and their eventual outcome. The results of our recent systematic review on the efficacy of steroids in patients with cardiac arrest suggested that supplementation with corticosteroids may improve the rate of survival and ROSC, especially in patients with hemodynamic instability and past medical history for cardiovascular disorders [34].

The results obtained from this meta-analysis are crucial, yet they need to be interpreted carefully. Although we tried to explore all the studies in this field, only five studies met the inclusion criteria for meta-analysis. In three of these studies, non-survivors of CPA not only did have low baseline cortisol levels, but the patients’ cortisol levels were even higher than normal [1, 11, 19]. Simultaneously, two clinical studies aligned to the results of this meta-analysis suggested a lower baseline cortisol level in non-survivors of CPA [17, 18]. In the study by Hékimian et al., 32 resuscitated patients after out-of-hospital cardiac arrest were prospectively evaluated for baseline cortisol and adrenal reserve after ROSC. The authors of this high-quality cohort concluded that early death following CPA may be associated with adrenal insufficiency and inadequate response of the adrenal cortex to this stressful event [11]. In another cohort study by Tavakoli et al., the serum levels of cortisol were assessed in 50 resuscitated OHCA patients after ROSC. In this prospective study, cortisol levels were significantly higher in those who neurologically survived than non-survivors [18]. Mosaddegh et al. investigated the clinical outcome of 52 IHCA patients over 3 months. Regarding the design of the study, although the authors used the term “clinical trial,” no intervention was made and patients were only evaluated for the relationship between serum cortisol levels following ACTH stimulation test and in-hospital mortality and discharge from the hospital. No significant difference was observed in baseline levels of cortisol between survivor and non-survivors of IHCA [19]. In the study by Ito, the possible link between the serum levels of stress hormones, including cortisol and the eventual outcomes of resuscitated patients after OHCA was investigated. In this study, with high quality and low risk of bias, the serum levels of cortisol were significantly higher in those who survived after CPA [17]. Finally, in the fifth study included in this meta-analysis by Kim et al., the prevalence of relative adrenal insufficiency following ROCS after cardiac arrest was evaluated. In this study, basal cortisol levels were measured in 30 patients as a secondary endpoint. Contrary to expectations, the basal levels of cortisol were normal or even high in patients experiencing CPA [1].

Unfortunately, the diversity of eligible studies for meta-analysis was high, and they did not have similar endpoints. These studies excluded the patients with unsuccessful CPR. Basal cortisol levels were not measured at all in the patients who did not effectively resuscitate. This may vigorously affect the taken results. The second major limitation of the present systematic review was the variation of the eligible studies for meta-analysis. As the significance between-study heterogeneity indicates, the included studies were inconsistent and heterogeneous. Moreover, the number of enrolled patients in these studies was limited so that the results of this meta-analysis were obtained from only 201 participants in total. This could justify the fact that the observed differences among the study groups did not reach a significant level, but still favored the lower basal cortisol in the non-survivors of CPA. Moreover, using therapeutic hypothermia is an effective intervention in CPA patients. Thus, this modality can act as a confounding factor on serum cortisol levels [35, 36]. This intervention was initiated in two studies of Hékimian et al. and Kim et al. in all admitted patients as early as possible by using wet and cold wraps, ice packs, and with neuromuscular blocking and continued for 24 h [1, 11]. Due to the small sample size in these studies, it was not possible to eliminate the effect of this confounder. High mortality in CPA patients can also affect the interpretation of mortality data.

## Conclusion

The current systematic review and meta-analysis is the only study which systematically covers all existing literature in this area to explore a relationship between baseline cortisol level and adrenal reserve with the outcome of patients resuscitated after CPA. The result of present meta-analysis was suggestive of higher baseline serum cortisol levels in survivors of CPA compared with non-survivors, although the differences between groups did not reach a significance level. Future randomized controlled studies with a large sample size will determine the exact relationship between adrenal reservation and the eventual outcome of patients with CPA.

## Data Availability

All data has been summarized and provided in the article. Subsequently, dataset generated through this systematic review can be requested from the corresponding author.

## Abbreviations

CPA: Cardiopulmonary arrest
HPA: Hypothalamic-pituitary-adrenal
RCTs: Randomized clinical trials
ACTH: Adrenocorticotropic hormone
PROSPERO: Prospective Register of Systematic Reviews
PI(E)CO: Patient, Intervention, (Exposure), Comparison, Outcome
MeSH: Medical Subject Headings
GRADE: Grading of Recommendations Assessment, Development and Evaluation
SE: Standard error
F/M: Female/male
ADH: Antidiuretic hormone
i.v.: Intravenous
ROSC: Return of spontaneous circulation
RAI: Relative adrenal insufficiency
